# Walking like an ant: a quantitative and experimental approach to understanding locomotor mimicry in the jumping spider *Myrmarachne formicaria*

**DOI:** 10.1098/rspb.2017.0308

**Published:** 2017-07-12

**Authors:** Paul S. Shamble, Ron R. Hoy, Itai Cohen, Tsevi Beatus

**Affiliations:** 1 Department of Neurobiology and Behavior, Cornell University, Ithaca, NY 14853, USA; 2 Department of Physics, Cornell University, Ithaca, NY 14853, USA

**Keywords:** Batesian mimicry, locomotion, spider, ant, ant mimicry

## Abstract

Protective mimicry, in which a palatable species avoids predation by being mistaken for an unpalatable model, is a remarkable example of adaptive evolution. These complex interactions between mimics, models and predators can explain similarities between organisms beyond the often-mechanistic constraints typically invoked in studies of convergent evolution. However, quantitative studies of protective mimicry typically focus on static traits (e.g. colour and shape) rather than on dynamic traits like locomotion. Here, we use high-speed cameras and behavioural experiments to investigate the role of locomotor behaviour in mimicry by the ant-mimicking jumping spider *Myrmarachne formicaria*, comparing its movement to that of ants and non-mimicking spiders. Contrary to previous suggestions, we find mimics walk using all eight legs, raising their forelegs like ant antennae only when stationary. Mimics exhibited winding trajectories (typical wavelength = 5–10 body lengths), which resemble the winding patterns of ants specifically engaged in pheromone-trail following, although mimics walked on chemically inert surfaces. Mimics also make characteristically short (approx. 100 ms) pauses. Our analysis suggests that this makes mimics appear ant-like to observers with slow visual systems. Finally, behavioural experiments with predatory spiders yield results consistent with the protective mimicry hypothesis. These findings highlight the importance of dynamic behaviours and observer perception in mimicry.

## 1. Introduction

Protective Batesian mimicry is a widespread phenomenon in which individuals of a palatable species avoid predation by being mistaken for an unpalatable model [1,2]. Often regarded as one of the finest and most convincing examples of adaptive evolution, this phenomenon is commonly exemplified by moths with the bright colours of a butterfly [3] or grasshoppers that seem every bit the shape of a tiger beetle [4]. Evolution in such mimetic systems involves three groups: mimics, models and predators, resulting in complex selective landscapes. Selection in these systems is expected to favour mimics that are increasingly similar to their models while also selecting for predators (often referred to as observers) that are increasingly able to discriminate between palatable mimics and unpalatable models. These selective forces are expected to result in mimics that exceedingly resemble their models [5]. However, naturalists in the field have long noted that many seemingly mimetic species appear to be only poor facsimiles of their models, a phenomenon referred to as imperfect mimicry [6]. Recent work on this topic has highlighted the importance of observer perception in shaping these interactions [6–9]. Thus, the ability to accurately quantify and compare traits across species is crucial to understanding the similarities—or perceived similarities—between mimics and models.

While studies of protective mimicry have overwhelmingly focused on static, rather than dynamic visual traits, the importance of motion has been part of mimicry theory since its inception. Bates noted in his foundational work that mimetic butterflies and their models were indistinguishable in flight [1]. Recently, studies on the neural mechanisms of visual behaviour have highlighted the importance of movements as behaviourally relevant cues [10–12]. However, only recently have researchers begun to rigorously investigate the dynamic, locomotor aspect of mimicry [13]. To date, research of this phenomenon has focused on two flight-based mimicry systems: passion-vine butterflies [13–15] and hymenopteran-mimicking hoverflies [16,17]. The study of locomotor mimicry among terrestrial mimics, meanwhile, has been limited [18,19].

**Figure 1.**
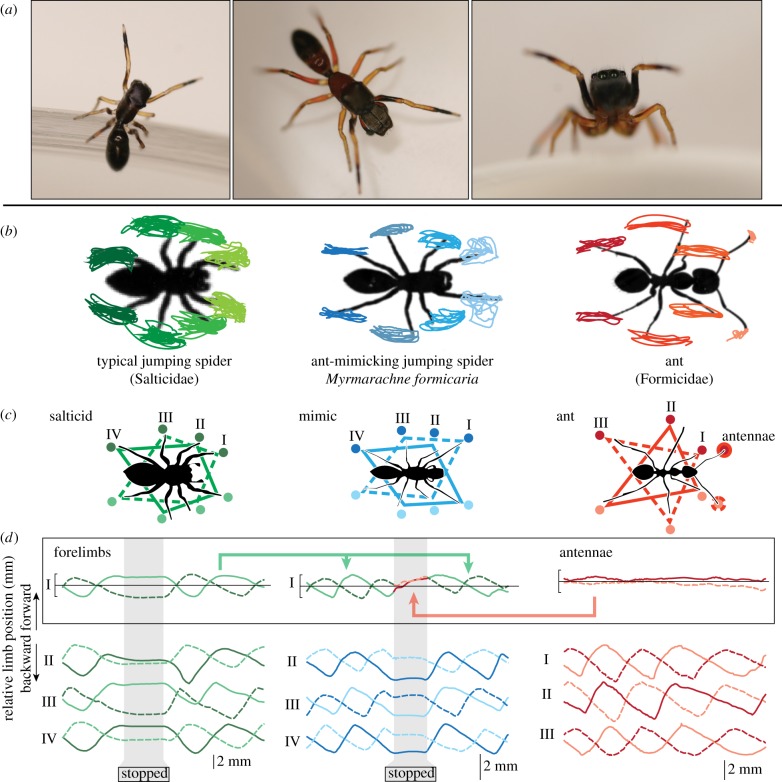
Gait analysis. (*a*) Images of an *M. formicaria* female (left) and a male (centre, right) showing the antennal illusion behaviour where the forelegs are elevated similar to ant antennae. A head-on view (right) showing the large anterior median eyes characteristic of jumping spiders. (*b*) The ends of appendages were tracked over multiple strides based on top-view high-speed videos. Tracking results from a single trial, superimposed over the animal silhouette in the reference frame of the animal. (*c*) The typical gaits of non-mimetic jumping spiders (left), mimetic *M. formacaria* (centre) and ants (right). Line style (dotted or solid) illustrates legs that move in-phase; these two sets then move in anti-phase. (*d*) Appendage positions plotted, forward/backward relative to the mean position over multiple strides. Colours indicate appendages on the left/right side of the body; dotted and solid lines as in (*c*). Non-mimetic jumping spiders show the typical alternating tetrapod gait and when stationary (grey zone) do not move their legs. Ants use an alternating tripod gait, with no clear phase relationship between antennae and legs. Ant mimics walk like typical jumping spiders (highlighted in green), but when stationary its forelegs move into phase with one another, similar to ant antennae (shown in red).

Within terrestrial mimicry, mimicry of ants (Hymenoptera, Formicidae) is among the most common [4], with spiders representing a large fraction of ant-mimicking species and occurring on nearly every continent [20–22]. In spiders, ant mimicry appears to have evolved dozens of times independently [21], with a recent thorough phylogenetic analysis of one family in particular, the jumping spiders (Araneae, Salticidae), suggesting 12 or 13 independent origins of ant mimicry in this group alone [23]. The *Myrmarachne* McLeay 1835 salticid genus (figure 1*a*) provides a spectacular example of ant mimicry, with over 217 described species [24]—virtually all of which are believed to be Batesian mimics [25]. Ants seem especially worth mimicking as they possess species-specific combinations of powerful defensive traits, including powerful mouthparts, venomous stings, chemical defences, general aggressiveness and the ability to recruit nest-mates [26]. Ants also tend to be highly conspicuous and abundant, further increasing their effectiveness as Batesian models [27].

Spiders, however, lack the specialized defensive traits of ants, perhaps most notably specialized chemical compounds. That said, spiders are formidable predators and are avoided by many species, especially would-be prey [28]. Jumping spiders are models of mimicry, themselves, with moth and tephridid fly species possessing leg-like wing patterns, which they wave aloft to recreate the arms-raised display used by jumping spiders to avoid potentially costly confrontations between individuals [29–31]. However, this only protects them from a subset of predators, with a range of species still considered to be important spider predators, including other spiders, nematodes, wasps, endoparasitic flies (Acroceridae), toads, lizards, birds and even some small mammals such as shrews [32]. In the context of ant mimicry, authors have suggested that cursorial spiders and wasps are particularly important because many are common predators of spiders, they possess relatively high-acuity vision and they show a preference for spiders over ants—exemplified by the many solitary parasitoid wasp species that specialize on spiders [20,21]. That said, other members of this list also possess high-acuity vision and a preference for spiders over ants—for example, among birds inhabiting European farmlands, arachnids ranked above hymenopterans in both the diets of adult birds during the breeding season and the diets of chicks [33]. Indeed, while the precise predators involved may vary, the general presence of predators that prefer spiders over ants establishes the selective landscape required to promote ant mimicry, providing a selective advantage to spiders that are mistaken for ants [20].

While the modifications required to make a jumping spider appear more similar to an ant might initially seem trivial, these taxa are separated by significant differences in morphology, behaviour and hundreds of millions of years of evolutionary history. Morphologically, mimicry is tasked with transforming a stocky arachnid with eight legs and two body segments into a thin insect with six legs, two antennae and three body segments with narrow constrictions (figure 1*a*,*b*). Behaviourally, the differences between the two are similarly formidable. Jumping spiders are solitary predators famous for visually driven behaviours [34,35]. They typically stalk their prey carefully, leaping towards their targets from many body lengths away. Ants, however, are social opportunistic foragers whose worlds are dominated by chemical cues [26]. With varying levels of cooperation (e.g. as solo foragers, tandem runners, recruited groups, etc.), ants wander the environment until they encounter a target, at which point they either recruit nest-mates to the site or collect the resource on their own and return it to the colony [26]. This transformation of spider to ant is also non-trivial at more mechanistic levels of behavioural output and motor control—while ants use opposing sets of extensor and flexor muscles to drive their legs and other appendages, spiders lack extensor muscles in major leg joints [36,37], instead using hydraulic pressure generated in their head to extend their legs [37,38]. Furthermore, while most examples of mimicry involve species of the same class or order—e.g. snakes mimicking snakes, or even moths mimicking butterflies—jumping spiders and ants are much more distantly related: members of completely separate subphyla (Chelicerata and Hexapoda), groups thought to have diverged approximately 540–600 Ma [39,40].

Locomotor mimicry of ants has often been obliquely invoked by researchers stating that ant-mimicking species *appear* to move like their models [21,41,42]. However, despite some attempts at quantification [18,19], this observation has remained largely qualitative [43]. For example, despite the lack of high-speed measurements of gait, it is often suggested that ant-mimicking spiders walk on six legs rather than eight [21,41,44,45]—a modification to the default gait that would seem to require significant changes to behavioural and locomotor neural underpinnings [46]. A main question thus remains: what does it mean to walk like an ant? That is, what traits of ant-mimic locomotion are ant-like and how they are perceived by potential predators?

Here, we sought a quantitative approach to understanding terrestrial mimicry by characterizing and comparing the locomotor traits of the ant-mimicking jumping spider *Myrmarachne formicaria* with those of ants and non-mimetic jumping spiders. To explore differences in limb use—or gait—we used multiple high-speed cameras to track leg movements of freely moving animals in three dimensions. We also compared overall movement patterns of animals through the environment, including the trajectories of ants following experimentally drawn pheromone trails, focusing our analyses on trajectory shape and temporal aspects of movement. Because the role of predator perception is central to the phenomenon of mimicry [9], we then conducted analyses exploring how the categorization of targets based on overall movement varies with the temporal resolution of the observer's visual system. Finally, in behavioural experiments, we presented large predatory jumping spiders with video animations of ants, mimics and non-mimetic jumping spiders to specifically ask whether these mimics incur reduced predation due to their visual similarities with ants. We discuss our results in the context of potential trade-offs faced by ant mimics and the possible role of other selective pressures, particularly predators. Overall, our findings highlight the importance of dynamic behaviours and predator sensory perception in the evolution of mimicry systems.

## 2. Material and methods

### (a) Animals

Animals were collected within 15 miles of Ithaca, NY, USA from April to August 2011 (gait studies) and June 2014 to July 2015 (overall movement studies). Spiders were housed in individual plastic containers under a 12 L : 12 D cycle at 23 ± 2°C, provided with a constant source of moisture, and sustained on fruit flies (*Drosophila melanogaster*) and/or domestic crickets (*Acheta domesticus*). Ants were used in experiments on the day of collection.

### (b) Gait analysis

To compare the gaits of ants, mimics and non-mimetic salticids, animals were filmed walking across a glass surface using three high-speed cameras, one top view and two orthogonal side views (Phantom v. 7.1, Vision Research), back-lit by red LEDs (Diamond Dragon, Osram Opto Semiconductors; peak wavelength ± spectrum width at 50% intensity = 625 ± 10 nm) (figure 1*a*,*b*). Animals moved freely across an 8 × 10 cm glass plate covered with clear plastic tape to improve traction. Video was captured at 1000–4000 frames per second, with a spatial resolution of 11.8 pixels mm^−1^; top-down fields of view = 9.44 × 7.08 cm^2^; side-view fields of view = 9.44 × 3.02 cm^2^.

Data processing and analysis was done using Matlab. To track animal body position and orientation in the top view, we generated a template of the body without the legs that was fit to each frame using three free parameters: *x-* and *y*-coordinates of the body's centre of mass (CM) and the orientation of the body's long axis. Body position was smoothed using a Savitzky–Golay filter (window size = 101 samples; polynomial order = 4), which was also used to determine body velocity. Limb tips were tracked in all camera views using code based on a study by Revzen [47], modified for side-view tracking. Leg height was normalized by the height of the animal at the most dorsal point on the head for each video sequence.

High-speed video results are based on 27 recordings of *M. formicaria* (three females and two males), 15 recordings of ants (four *Formica* sp. workers) and 23 recordings of non-mimetic salticids (*Salticus scenicus* two females; *Sitticus* sp. one female; *Phidippus audax* two females).

### (c) Overall movement

Animals were allowed to move freely across a featureless arena (75 cm diameter) of white poster board, surrounded by a 60 cm high brown paper baffle to limit extraneous visual stimuli. Trials were recorded using a GoPro Hero 3+ Black camera (1280 × 720 pixel resolution; 120 frames per second), centred 80 ± 7 cm above the arena floor and calibrated using the Matlab Camera Calibration Toolbox. The arena was illuminated by two LED lamps (Utilitech, 6.5 W, 450 lumens, warm white) and standard laboratory lighting. Animals were introduced into the centre of the arena from below via an elevator made of a modified 60 ml plastic syringe fit into a hole in the arena floor. Animals acclimated inside the elevator for at least 5 min before the first trial, and 2 min between trials. Trials began when the animal left the elevator and concluded once it reached the arena boundary. Individuals were tested up to five times. Between trials, the arena was wiped down with 70% EtOH to remove deposited silk or chemical cues. Analysis is based on recordings of *M. formicaria*, the non-mimetic salticid *S. scenicus* and two ant species, *Lasius* sp. and *Tetramorium* sp. Full sample sizes for these experiments, including overall times and distances each animal type was observed, are detailed in electronic supplementary material, table S1.

To determine animal CM in each image, we segmented the darker animal against the background using binary thresholding and calculated the centroid of the pixels. The *x-* and *y-*coordinates were smoothed using a Savitzky–Golay filter (window size = 41 samples; polynomial order = 5), which was also used to determine velocity. Animals were deemed stationary if their velocity was below 4 mm s^−1^. To analyse trajectory shape, we removed stationary periods from trajectories, then equally sampled, spatially, at 0.2 mm intervals using cubic splines to produce a length parametrization without stops and velocity. Trajectory angle with respect to the laboratory *x-*axis was calculated by 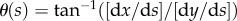, where *s* is the length parameter along the path. Curvature was defined as *κ*(*s*) = d*θ*/d*s*.

To quantify the regularity of each trajectory shape, we calculated its direction autocorrelation function:2.1
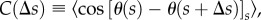
where *θ*(*s*) is the angle between the *x-*axis in the laboratory frame and the vector tangent to the trajectory measured at distance *s* along the path. The correlation *C* at a given relative path length Δ*s* is the cosine of the change in *θ* averaged over all point pairs that are separated by path length Δ*s*. Thus, *C*(Δ*s*) is a translational and rotational invariant function that captures the periodicity and amplitude of angular changes in a trajectory.

### (d) Trail following

To observe ants engaged in trail following, we extracted pheromones from ant abdomens [48] and used these extracts to draw trails. Ants observed and those used for extraction were collected from the same colony/location. To prepare extractions, ants were sacrificed in a freezer, and then abdomens were removed and placed in solvent (10 µl/abdomen) at room temperature for at least 10 min. Solvents used were hexane for *Tetramorium* ants [48], and acetone for *Lasius* ants.

The extract was loaded into a 20 µl pipette tip with a cotton wick fitted in the narrow end and drawn onto a piece of Carson art paper. Trails were approximately 2 mm wide and formed a 45 × 35 cm rectangle with rounded corners, consisting of 400 µl of extract. Trials lasted approximately 15 min, or until the ant left the arena. As a control, we tested the response of ants to solvent-only trails and observed no response. Video and tracking data were collected and analysed as described above. For trajectory autocorrelation function analysis, we considered only the straight segments of the rectangle, excluding the corners as they distort the analysis. Autocorrelations of each straight segment were treated independently. Ant activity before contacting the trail or when ants ignored the trail was considered off-trail and binned with the previously described trials of ants on featureless arenas.

### (e) Predator responses to playback animations

**Figure 2.**
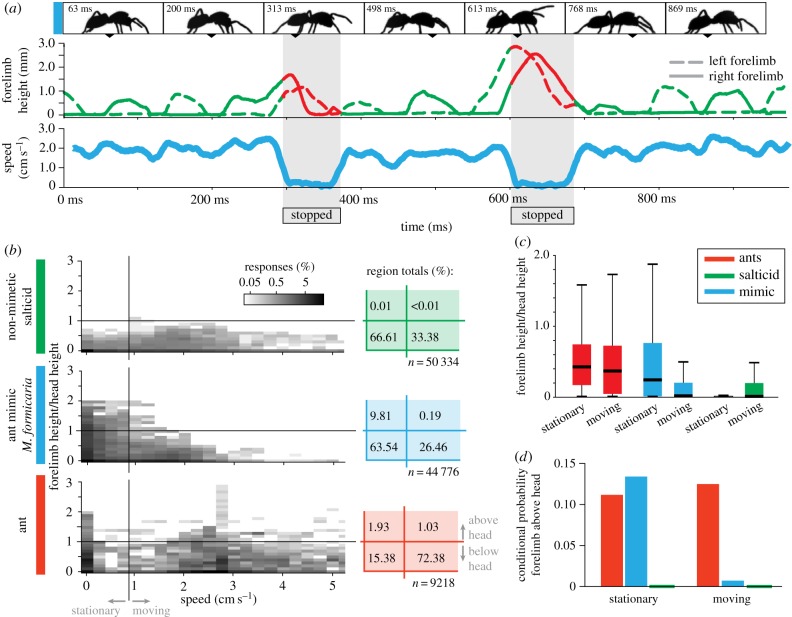
Forelimb height during locomotion. (*a*, top) High-speed video frames showing *M. formicaria* walking; elevated forelegs can be seen in the third and fifth images when the animal was stationary. (*a*, bottom) Foreleg height and animal speed for a single trial. When walking, forelegs move in anti-phase (green). When stopped, the forelegs are raised and move in-phase (red). Grey area highlights when the animal was stationary. (*b*) Probability density maps (log scale) for observing an animal with a given forelimb height and walking at a given speed (cut-off at 52 cm s^−1^ for clarity), over multiple high-speed videos (see Material and methods). Ant antennae were considered as forelimbs. Forelimb height is normalized by animal head height. Stationary/moving division set to less than 1 cm s^−1^. Total probabilities for the four regions (stationary/moving, limb above/below head height), shown at right highlighting mimic foreleg elevation when stationary. (*c*) Boxplot showing relative forelimb height for animals while stationary or moving. Centre lines denote medians, boxes extend to 25th and 75th percentiles, and whiskers to 99th percentiles of a normal distribution. Outliers beyond this range were excluded for clarity. All groups are significantly different from one another (Kruskal–Wallis; 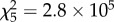; *p* < 0.001 after Bonferroni correction). (*d*) Conditional probability that forelimbs are above head height given an animal is either stationary or moving. Salticid values (green) are shown as a line with weight for visibility—both values are positive but <0.001.

To experimentally test the predictions of Batesian mimicry using playbacks and real predatory observers, we focused on the large jumping spider, *P. audax*, a generalist, visual predator of small arthropods. Our experiments explored how potential prey body shape and limb movement (figure 2) influence attack behaviour.

Mimic, ant and non-mimic salticid animations (electronic supplementary material, videos S1–S3) were based on our high-speed side-view videos, resampled so targets appeared to move normally when played at 30 frames s^−1^. Body length (5 mm), velocity (approx. 1.5 cm s^−1^) and overall movement were identical across targets. Distinct body shapes were generated for each target type (figure 6*a*). Mimic targets also retained the abdomen-bob behaviour characteristic of *M. formicaria*.

Targets varied specifically regarding forelimb behaviour (figure 6*a*), treating ant antennae as forelimbs. Consistent with our video observations, ant target antennae were elevated when moving, tapping the ground once as the target stopped; *M. formicaria* target forelimbs were raised when stationary and operated as legs when moving (antennal illusion, figure 2); and non-mimetic salticid target forelimbs operated as legs throughout, remaining down when the target was stationary. The remaining six legs were identical across targets. Each target made 24 total passes across the screen, with one 1.5 s pause at the centre and six 0.1 s pauses, spaced roughly equally across the screen. The total video duration was 3 min 20 s.

For each trial, predators were lured onto a trapezoidal platform (parallel sides 7.5 cm and 21 cm, length 25 cm) using bait to ensure that they were behaviourally motivated to hunt. Animations were presented on a high-resolution LCD screen (Apple iPhone 5 s; 7.5 × 5.0 cm; image 960 × 640 pixels) at the opposite end of the platform. We then observed whether the spider pounced at the target (electronic supplementary material, video S4). Each *P. audax* (*n* = 12) experienced each target once, with order varied between individuals.

### (f) Statistical tests

Given the prevalence of non-parametric data, Kruskal–Wallis tests were used for multiple comparisons. The Mann–Whitney *U* test was used when comparing between two groups that were not normally distributed. All *p*-values reported are after Bonferroni correction.

## 3. Results

### (a) Limb kinematics

Top-view measurements confirmed known gaits of ants [49] and non-mimetic jumping spiders [45] (figure 1*b–d*): alternating tripods (figure 1*b–d*, right) and alternating tetrapods [50] (figure 1*b–d*, left), respectively. Antennae were constantly held aloft and in front of the body. *Myrmarachne formicaria* moved—exclusively—on eight legs, across all 58 observed forward-moving steps (figure 1*b–d*, centre). However, when stationary, the forelegs were typically brought into phase and extended upwards and forwards—generating the antennal illusion behaviour characteristic of many ant-mimicking spiders [21,41,51] (figure 1*d*, centre). Stationary episodes were approximately 100 ms (mean stop duration ± standard deviation 83.3 ± 82.6 ms; *n* = 41). Figure 2*a* shows two antennal illusion events, illustrating the anti-phase motion of forelimbs while moving and the simultaneous forelimb elevation when stationary.

To compare forelimb height and animal speed, we plot a two-dimensional probability distribution of these quantities for each animal type (figure 2*b*). Axes are divided into regions: stationary/moving regarding speed (threshold of 1 cm s^−1^) and above/below head height along the forelimb height axis. While forelimb height of non-mimetic spiders and ants was largely insensitive to animal speed, mimics showed a distinct pattern of increased forelimb height while stationary, summarized by the probability integrated over each of the four regions (figure 2*b*, right) and by normalized height (figure 2*c*). Differences between mimics and the other animal types are even more pronounced when considering the conditional probability for observing forelimb above the head height given the animal speed category (figure 2*d*). When moving, mimic forelimb behaviour matches that of non-mimetic jumping spiders, but when stationary, mimic forelimb behaviour is similar to that of ant antennae.

### (b) Overall animal trajectories

**Figure 3.**
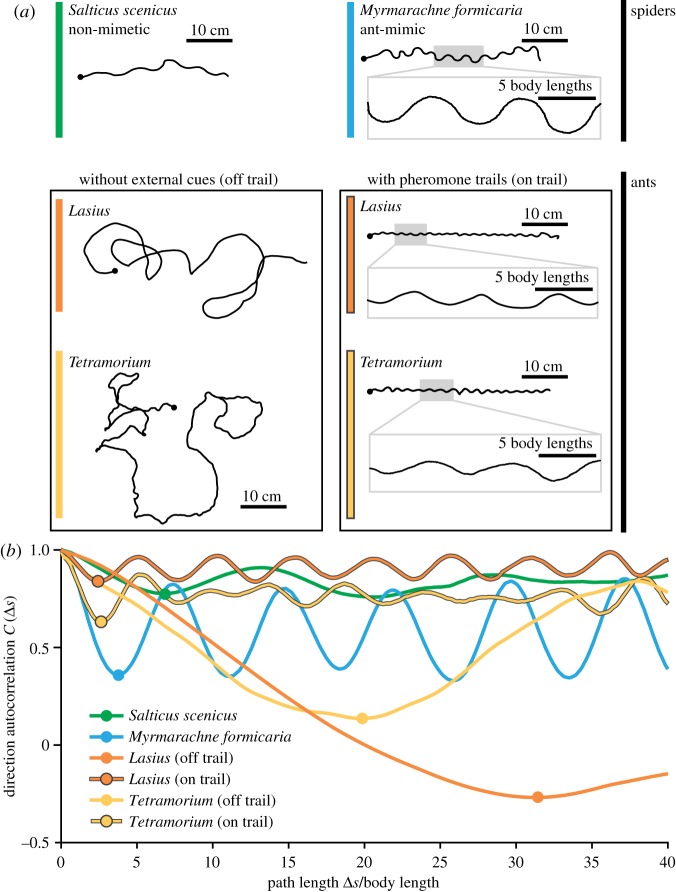
Overall trajectory analysis. (*a*) Example traces of paths walked by a non-mimetic jumping spider (*S. scenicus*), an ant mimic (*M. formicaria*) and two ant species. All spiders were tracked moving across a featureless arena; ants were on a featureless arena or following a pheromone trail. Black dots mark the starting position of each trajectory. Grey boxes show a portion of the trail at higher magnification. (*b*) Direction autocorrelation *C*(Δ*s*) for each example shown in (*a*). Circles on each line mark the first autocorrelation minimum, *C*(Δ*s*_min_).

Example overall trajectories are shown in figure 3*a*. Mimic trajectories were characterized by curved, regular, sine-like shapes (typical wavelength approx. 10 body lengths; amplitude approx. five body lengths). Non-mimetic spider trajectories were less curved, less regular and less periodic. Movement of ants on featureless arenas was characterized by large, looping trajectories, a hallmark of ants engaged in searching [52]. However, ants following trails walked highly regular sine-like routes (typical wavelengths approx. 10 body lengths; amplitude 2–5 body lengths). These oscillatory trajectories are a functional consequence of how ants navigate along pheromone trails; tacking across the chemical trail until the signal becomes sufficiently weak, then tacking back in the opposite direction [53]. Importantly, all spider trajectories (mimics and non-mimics) were measured on featureless arenas lacking such trails.

Autocorrelations of trajectories shown in figure 3*a* are plotted in figure 3*b* as a function of path length normalized by animal body length. Autocorrelations for both salticid and ant species in the trail-following condition are highly periodic, indicating an oscillating direction of motion. However, autocorrelations for ant trajectories on featureless arenas lack such periodic structure. These differences can be quantified through measurement of the position, Δ*s*_min_, and value, *C*(Δ*s*_min_), of the first local minimum of the autocorrelation. Intuitively, length 2Δ*s*_min_ corresponds to the oscillation wavelength along the trajectory, while the value *C*(Δ*s*_min_) corresponds to the cosine of the average angular deviation for half an oscillation cycle, with lower values indicating larger angular deviation. These measures clearly differentiate the looping trajectories of ants off-trail (large Δ*s*_min_, small *C*) from the highly curved and regular trajectories of ants on trails and mimics (small Δ*s*_min_, large *C*) and capture the relatively straighter trajectory shapes of non-mimetic salticids (figure 3*b*).

**Figure 4.**
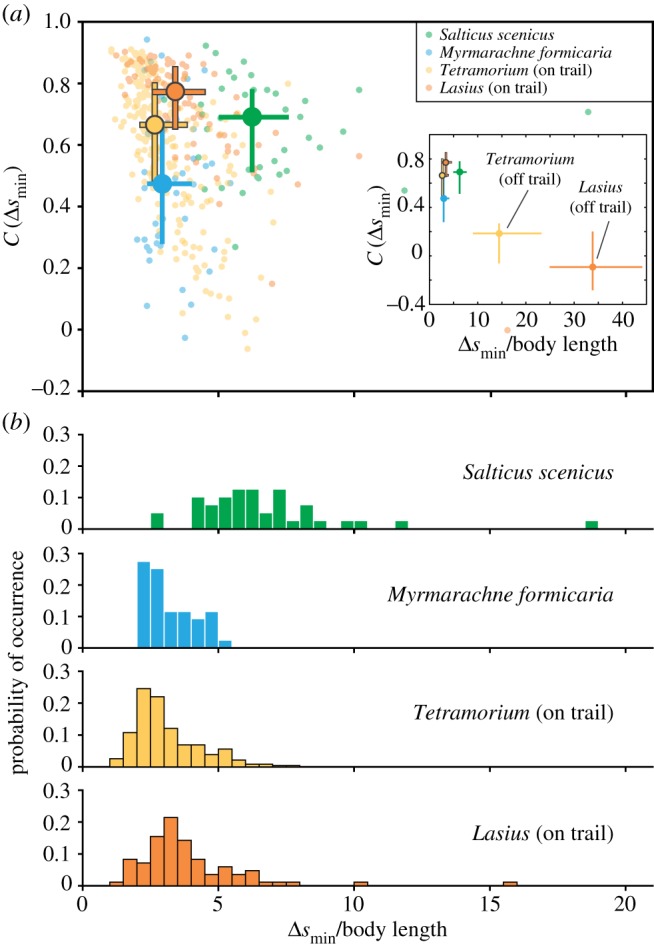
Comparative trajectory analysis. (*a*) Scatterplot showing first autocorrelation minima *C*(Δ*s*_min_) (figure 3*b*). Each point represents an animal trajectory. For ants following trails, points represent a straight segment of a trajectory. Minima position, Δ*s*_min_, was normalized with respect to the body length. Cross centre indicates the median; arms extend to ±25% of the distribution. Comparing Δ*s*_min_ across mimics, on-trail ants and non-mimetic jumping spiders (Kruskal–Wallis; 
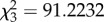
); mimics are indistinguishable from on-trail ants (*p* > 0.3); differences between on-trail ant species are significant (*p* < 0.001), and non-mimetic jumping spiders are significantly different from the other groups (*p* < 0.0001). Inset is rescaled to show all animal conditions, including ants off-trail. (*b*) Probability distributions of the normalized Δ*s*_min_ values for the four conditions shown in (*a*), highlighting the similarity between mimetic jumping spiders and ants on-trail.

To elucidate geometric differences between animal trajectories, we plot *C*(Δ*s*_min_) versus Δ*s*_min_ across the entire dataset (figure 4*a*). Cross centres in figure 4*a* mark medians of *C* and Δ*s*_min_ for each population, and cross arms cover 25th–75th percentiles. Histograms in figure 4*b* show the probability distribution of Δ*s*_min_. Statistical comparisons (Kruskal–Wallis; 
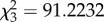
) reveal that Δ*s*_min_ values for mimics, and both on-trail ant groups are significantly different from non-mimetic salticids (*p* < 0.0001). Although both ant species while on-trail were significantly different from one another (*p* < 0.001), mimics and ants on-trail were statistically indistinguishable (*p* > 0.3).

**Figure 5.**
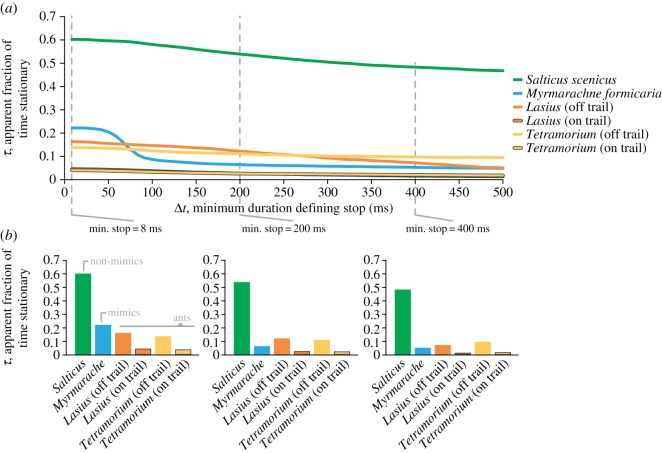
Temporal trajectory analysis as a function of the observer's CFF. (*a*) The fraction of time each animal appears stationary, *τ*, versus the duration of the shortest perceivable stop Δ*t*. Δ*t* is a property of the visual system of a potential observer, estimated as the reciprocal of its CFF. (*b*) Cross sections at the three stop-duration values denoted by the dotted grey line are shown to highlight how an observer with a given set of visual capabilities might categorize the movements of each group.

Temporally, the trajectories of ants and regular salticids represent two extremes of locomotory dynamics: ants move almost constantly while non-mimetic salticids often display long stationary periods. It is therefore informative to ask what fraction of time mimetic spiders spend stationary and how potential predators might use this information to classify potential targets. Determining whether an object is stationary, however, depends on the observer's visual temporal resolution, related to its critical flicker fusion frequency (CFF) [54,55]. Visual systems with higher CFF values can detect shorter stops, increasing the *apparent* fraction of time a target animal appears stationary. To quantify how variation in temporal resolution influences *perceived* locomotor behaviour of the observed species, we calculated *τ*, the fraction of time each animal appears stationary, versus the duration of the shortest perceivable stop Δ*t* (figure 5).

Non-mimetic salticids are often stationary (*τ* ∼ 0.55), while ants both off-trail (*τ* ∼ 0.1) and on-trail (*τ* ∼ 0.05) are not. Non-mimetic salticids and ants show a weak dependence of *τ* on Δ*t*. Mimics, however, show a sharp decrease from *τ* ≈ 0.22 to *τ* ≈ 0.09 between Δ*t* of 50 and 100 ms, indicating that most stops are between 50 and 100 ms long. Consequently, to observers with lower CFF values (approx. 10 Hz), mimics would appear to move almost constantly, similar to ants. Similar results are observed in measurements of the distance and time between consecutive stops (electronic supplementary material, figure S1).

### (c) Behavioural responses to playback animations

**Figure 6.**
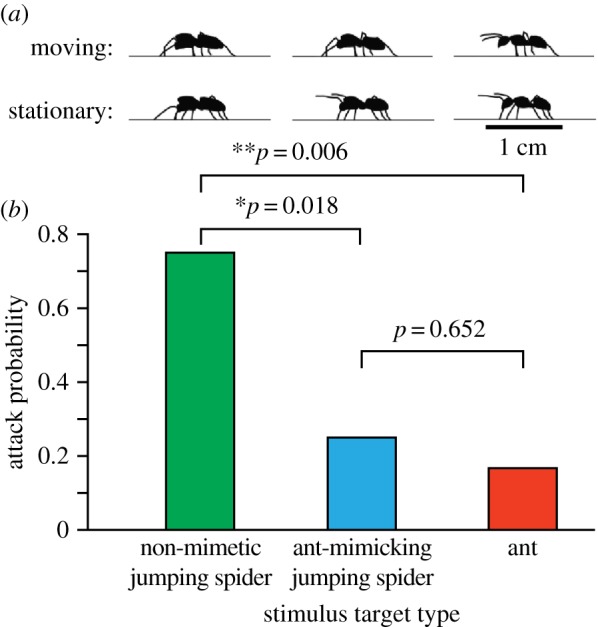
Behavioural playback experiments. (*a*) Snapshots of the three target types used in the playback experiments. (*b*) The probability of observing a predator attack based on target type. Predators attacked non-mimic targets significantly more than the other target types and showed no difference in attack probability between ant mimic and ant targets (Wilcoxon each-pair test).

We observed a significant effect of target type on attack (logistic fit, general linearized model; *χ*^2^ = 6.086, *p* = 0.0477), but no significant effect of presentation order (*χ*^2^ = 8.2 × 10^−8^, *p* = 1), or the interaction term (*χ*^2^ = 6.727, *p* = 0.1511). Non-mimetic jumping spider targets were attacked 4.5 times more than ant targets (Wilcoxon test; *p* = 0.0055) and three times more than mimic targets (*p* = 0.0180), and there was no significant difference between the number of attacks on ants and mimics (*p* = 0.6520; figure 6; electronic supplementary material, figure S2)—results consistent with protective Batesian mimicry.

## 4. Discussion

Our results quantitatively show how the movement of the ant-mimicking jumping spider, *M. formicaria*, is similar to that of ants both at short, single-step timescales and at long, full-trajectory timescales. At short timescales, and at the level of limb kinematics, mimics move using all eight legs like other spiders, a result contrary to the widely held belief that ant-mimicking spiders walk on six legs [21,41,44,45]. However, mimics also perform short, approximately 100 ms stops, when they exhibit an antennal illusion behaviour [21,41,51]. Regarding overall motion, the mimics' sine-like trajectories and their propensity for continuous movement are similar to ants specifically engaged in trail following. Indeed, although these mimics accurately imitate the zig-zag behaviour of ants, they reveal a form of contextually imperfect mimicry by producing this behaviour even in settings where ants do not. Furthermore, while the approximately 100 ms pauses made by *M. formicaria* appear brief enough to challenge the visual systems of many species (including humans) to determine when these animals are stationary, some observers (e.g. various birds) may possess sufficiently fast visual systems to detect such pauses, potentially helping them categorize mimics as jumping spiders (see [54] for a review of CFF values across species). Finally, behavioural experiments demonstrate that predator responses in this system are consistent with protective Batesian mimicry.

That these ant mimics do not walk on six legs is especially interesting in the context of locomotion in other arachnids. Although all arachnids are eight-legged, multiple orders locomote on six, with one pair having become specialized sensory antennae-like structures (e.g. amblypigids (Amblypygi), vinegroons (Thelyphonida) and opilionids (Opiliones) [45]). Even among spiders six-legged locomotion is possible, although most often a result of limb autotomy due to confrontations with predators [56]. This leads one to wonder if the eight-legged movement of *M. formicaria* represents a limit to the malleability of the neural circuitry governing normal locomotion, or insufficient time or selective pressure. High-speed, quantitative studies of gait in other ant-mimicking spiders might help to address this. If other ant-mimicking spiders walk on six legs, this would suggest that the specific selective pressures are responsible for the presence or absence of this trait, rather than constraints present across the lineage. However, the six-while-stationary strategy described here might simply be sufficient to successfully modify operator behaviour—in line with the established eye-of-the-beholder hypothesis concerning imperfect mimicry [6]. This seems especially possible given the role of predator perception and cogitation in mimicry [9]. For example, if observers best discern target details when targets are stationary, they might weight this information more heavily—a time when mimic forelimb behaviour is accurately ant-like.

### (a) Trade-offs due to ant mimicry

Given that *M. formicaria* evolved from non-mimetic jumping spider ancestors [23], the observed differences in locomotor behaviour leads to the question of potential costs associated with the evolution of ant mimicry. Regarding overall movement, the continuous, winding movements of these mimics seem potentially non-optimal, probably increasing metabolic cost and exposure to predators compared with the more direct, punctuated locomotion of non-mimetic jumping spiders. Interestingly, the trajectories of non-mimetic jumping spiders do show traces of this winding motion, suggesting that this feature of *M. formicaria* locomotion may have evolved from selection on an already present behaviour rather than representing a completely novel trait. Second, while typical jumping spiders leap on their prey from multiple body lengths away [57,58], *M. formicaria* and other ant-mimicking spiders lunge at targets from close range (electronic supplementary material, video S5) [42], suggesting a broad change in foraging strategies. Furthermore, over the course of our work, we did not witness *M. formicaria* definitively jump—a reluctance or inability that seems counter to one of the most fundamental innovations of the Salticidae family. If the loss of these typical jumping spider behaviours among ant mimics does indeed make them less efficient predators compared with non-mimics, then the repeated evolution and worldwide distribution of ant mimics makes a strong case for the significant anti-predator fitness benefits associated with ant mimicry.

However, it is also possible that these changes in locomotor behaviour have enabled ant-mimicking jumping spiders to invade new niches. While additional benefits of ant mimicry beyond Batesian protection have been suggested for spiders, these are typically associated with potential advantages of living in close proximity to ants [59], rather than changes linked to locomotor behaviour. Given the broad functional role of locomotion—finding prey and mates, mediating exposure to predators and in the context of mimicry acting as a cue that influences observer decision-making—selection for one aspect may influence others. For example, the winding trajectories of mimics might facilitate a switch in diet, with mimics trading weary prey that must be carefully stalked for prey that prefer to remain hidden and are therefore best hunted by increasing the search area covered. That is, mimics may have traded a target-oriented jumping spider-like foraging strategy for one more like the opportunistic foraging of ants. In this case, the worldwide distribution of ant mimics might instead represent repeated successful invasions of new niches, facilitated by positive feedback between mimicry-based ant-like behaviours and access to novel prey through ant-like foraging strategies.

### (b) Potential selective forces

We suggest that the multifaceted mimicry reported here may be a result of selective pressures imparted by multiple predator types, with specific predators potentially responsible for the evolution of specific mimetic traits. For example, a visually unobstructed and elevated vantage point seems crucial to enabling a predator to observe the curved trajectories of mimics and of ants following trails. The antennal illusion behaviour, however, seems likely to be most easily perceived from up-close—a view that a larger predatory jumping spider, a toad or a wasp might typically have just before attack. Furthermore, temporal aspects of the overall movement patterns of mimics seem particularly capable of fooling observers with slower visual systems, such as reptiles, amphibians and other jumping spiders versus predators with more rapid visual systems [54]. Specifically, if an observer is unable to resolve the short (approx. 100 ms) pauses made by mimics, these targets would appear to move in a nearly continuous manner similar to ants and highly unlike the motion of jumping spiders. Our behavioural playback experiments also provide initial support for this multi-perspective hypothesis by demonstrating that even a specific subset of mimetic traits is sufficient to influence predator behaviour. From these behavioural experiments, it is difficult to differentiate whether the predatory jumping spiders viewed the raised limbs of the ant and mimic animations as representative of the arms-raised display often used by jumping spiders to warn off other jumping spiders or of the raised antennae of an ant. Determining the precise cognitive effect of this cue on these predators would be a fascinating future direction—for example, both displays could simply signal the presence of unprofitable prey with no unique distinction between groups. Regardless, the functional result is the same in that ant and mimic targets were similarly avoided relative to non-mimetic targets—a result consistent with Batesian mimicry.

Indeed, multi-predator and multi-trait selective landscapes could be quite rich. It will be interesting to explore how specific traits influence the behaviours of different classes of predator, particularly when traits are tested in combination. One could also imagine that different predators could use the same traits to classify targets but with predator-specific weightings, or even that selective pressures from different predators could oppose one another for a given trait. Building on the quantitative approach taken here, it would also be interesting to survey the dynamic traits of other ant-mimicking species, particularly those that represent independent evolutions of ant mimicry, to determine the degree to which mimics use similar mimetic traits to achieve ant-like forms and whether such similarities or differences speak to shared predators. Finally, this quantitative understanding of traits should make it possible to experimentally generate trait values that are intermediate between non-mimics, mimics and models, work that could reveal how mimetic systems evolved and how they stabilize over time.

## Supplementary Material

Table S1, Figure S1 and Figure S2

## Data accessibility

All relevant data have been deposited online and are available from the Dryad Digital Repository at: http://dx.doi.org/10.5061/dryad.fd612 [60].
