# Supplementary material for: Walking like an ant: a quantitative and experimental approach to understanding locomotor mimicry in the jumping spider Myrmarachne formicaria
Source: Proc Biol Sci. 2017 Jul 12;284(1858):20170308. doi: 10.1098/rspb.2017.0308 (PMC5524487; doi:10.1098/rspb.2017.0308)
Supplement: Table S1, Figure S1 and Figure S2 [file rspb20170308supp1.docx]

**Supplemental Information**

**To walk like an ant: quantifying locomotor mimicry in the jumping spider *Myrmarachne formicaria***

Paul S. Shamble, Ron R. Hoy, Itai Cohen, and Tsevi Beatus

**Contents:**

**Table S1**. Sample sizes for overall animal trajectory experiments.

**Figure S1.** Analysis of animal trajectories as a function of the observer’s critical flicker fusion frequency (CFF).

**Figure S2.** Behavioral responses of the predatory jumping spider *Phidippus audax* to animated playbacks.

**SI Video S1.** Animation of ant target used in behavioral playback experiments.

**SI Video S2.** Animation of mimic target used in behavioral playback experiments.

**SI Video S3.** Animation of non-mimetic jumping spider target used in behavioral playback experiments.

**SI Video S4.** Example of a behavioral playback experiment with the large predatory jumping spider, *Phiddipus audax*, responding to a movie of a non-mimetic jumping spider-like target.

**SI Video S5.** Example of a male *Myrmarachne formicaria* lunging towards and capturing a fruit fly.

**Table S1. Sample sizes for overall animal trajectory experiments.**

| **species and condition** | **number of animals** | **number of sections** | **time observed (s)** | **distance observed (cm)** |
| --- | --- | --- | --- | --- |
| *Salticus senicus* | 9 | 51 | 2856.9 | 2448.5 |
| *Myrmarachne formicaria* | 9 | 82 | 3260.5 | 4768.1 |
| *Lasius* sp. | 26 | 152 | 4672.5 | 9451.3 |
| *Lasius* sp. on trail | 3 | 3 | 1584.5 | 4266.3 |
| *Tetramorium* sp. | 14 | 33 | 843.1 | 875.7 |
| *Tetramorium* sp. on trail | 11 | 11 | 8080.9 | 10109.7 |
| **TOTAL:** | **72** | **332** | **21298.4** | **31919.6** |


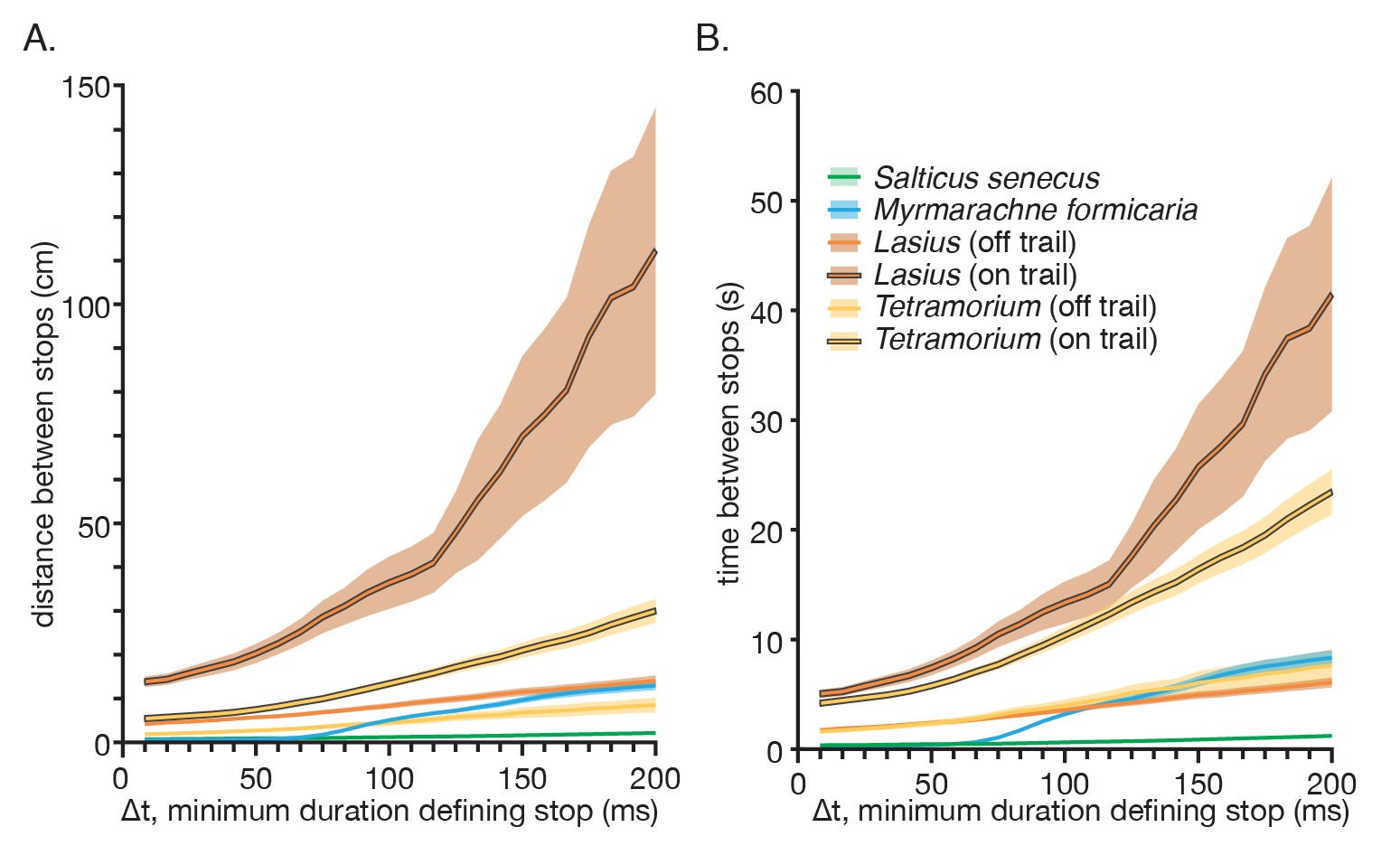


**Figure S1. Analysis of animal trajectories as a function of the observer’s critical flicker fusion frequency (CFF).** Related to Figure 5. The perception of how a target appears to move is dependent on the shortest perceivable stop $(\Delta t$) that the observer’s visual system can register. Above are **(A)** the apparent distance traveled by a target between consecutive stops and **(B)** the time elapsed between consecutive stops, both plotted as a fuction of $\Delta t$. Solid lines show mean values, shading shows the standard error of the mean. Colors are as indicated.


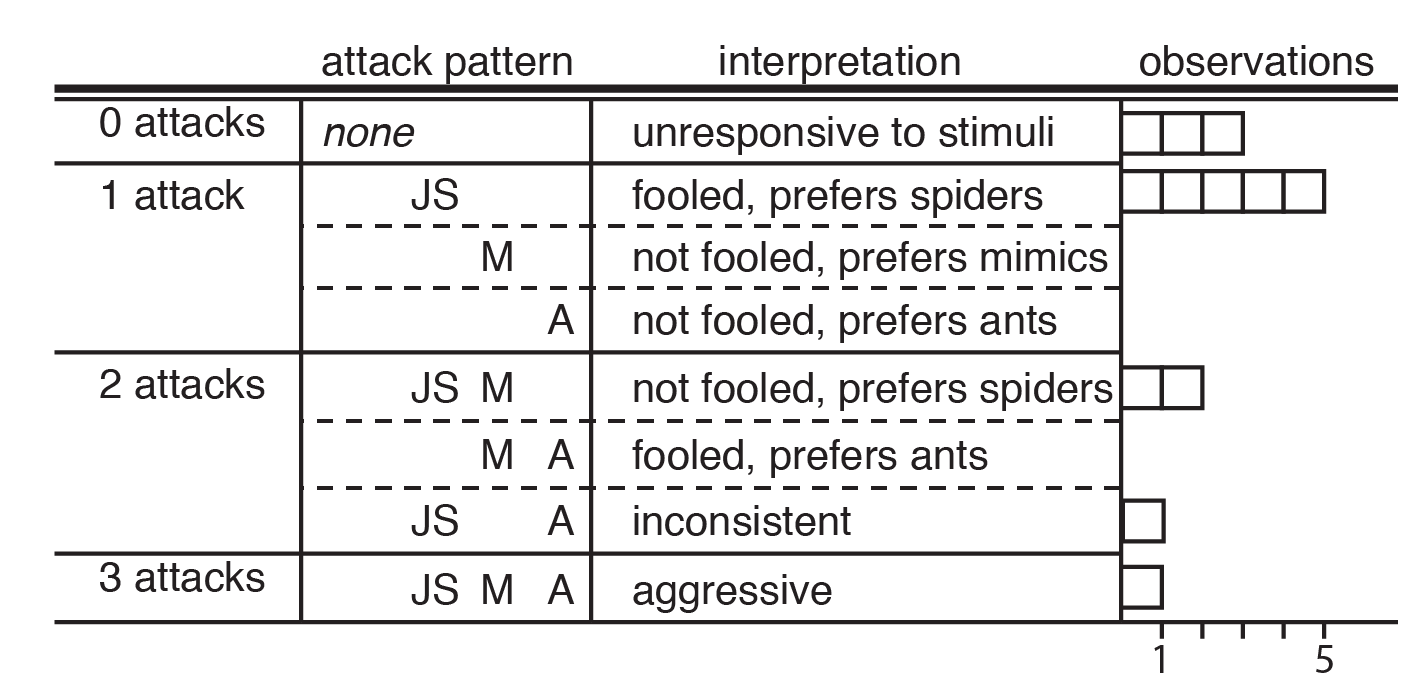


**Figure S2. Behavioral responses of the predatory jumping spider *Phidippus audax* to animated playbacks.** Related to Figure 6. Categorization of predator behavioral responses, with interpretations of results in the context of mimicry. Of the predators that attacked a target, the majority showed behavior consistent with Batesian mimicry, that is they only attacked the non-mimetic jumping spider (JS) target.

**SI Video S1.** Animation of ant target used in behavioral playback experiments.

**SI Video S2.** Animation of mimic target used in behavioral playback experiments.

**SI Video S3.** Animation of non-mimetic jumping spider target used in behavioral playback experiments.

**SI Video S4.** Example of a behavioral playback experiment with the large predatory jumping spider, *Phiddipus audax*, responding to a movie of a non-mimetic jumping spider-like target.

**SI Video S5.** Example of a male *Myrmarachne formicaria* lunging towards and capturing a fruit fly.
